# Hydrophilic Composites of Chitosan with Almond Gum: Characterization and Mechanical, and Antimicrobial Activity for Compostable Food Packaging

**DOI:** 10.3390/antibiotics11111502

**Published:** 2022-10-28

**Authors:** Raja Venkatesan, Surya Sekar, Chaitany Jayprakash Raorane, Vinit Raj, Seong-Cheol Kim

**Affiliations:** 1 School of Chemical Engineering, Yeungnam University, Gyeongsan 38541, Korea; 2 Department of Chemistry, College of Engineering Guindy, Anna University, Chennai 600025, Tamil Nadu, India

**Keywords:** chitosan, almond gum, mechanical strength, micro-structure, antimicrobial activities

## Abstract

To enhance the characteristics of the biocomposite film, solution cast was used to incorporate almond gum at different concentrations (10.0, 30.0, and 50.0%). The functional groups and morphology were determined using FTIR and SEM. The thermal property of chitosan and its composites materials were determined via TGA. In this study, the incorporation of almond gum into the chitosan matrix resulted in good mechanical strength, film thickness, and low barrier and solubility characteristics. Water vapor transmission rate (WVTR) and oxygen transmission rate (OTR) of the composites films was also investigated. The WVTR and OTR values for the chitosan/almond gum (CSA) composite film values are 11.6 ± 1.62 (g/m^2^/day) and 32.9 ± 1.95 (cc/m^2^/24 h), respectively. The obtained composites show significantly improved antimicrobial activity against Gram-negative (*E. coli*) and Gram-positive (*S. aureus*) food-borne pathogenic bacteria. The results suggest that the CSA composites may serve as a promising candidate for antimicrobial food packaging materials. After an observation of the test results, it is inferred that the CSA composites bear good mechanical and antimicrobial activity and also show enhanced morphological characteristics.

## 1. Introduction

Packaging is an important factor in the food industry and is dominated by petroleum-derived polymers. However, an abundance of research is now being conducted on the development and evaluation of biodegradable materials, mainly due to concerns over potential damage posed by synthetic packaging materials. Several biopolymers have been exploited to develop eco-friendly food packaging materials [1,2,3,4,5]. A significant proportion of research on these films has been performed using biopolymers from renewable sources, i.e., products or by-products derived from agriculture or from agro industries [6]. In a majority of cases, the biopolymer-based material shows less mechanical strength and is highly dependent on the environment [7]. As a result, several researchers have developed films based on blends of biopolymers and synthetic polymers. Several studies have been conducted in the preparation of bio-based polymers using hybrid materials [8,9,10].

Chitosan (CS) is a polysaccharide compound comprising an arbitrarily dispersed b-(1-4) conjugated D-glucosamine (deacetylated unit) and N-acetyl-D-glucosamine (acetylated unit). It is an outstanding biopolymer owing to its nontoxicity, biodegradability, biocompatibility, and bioactivity, giving it good film-forming characteristics. However, its mechanical and barrier properties are not suitable for food packaging applications, although it can be mixed with nanoparticles or other polymers to improve its properties. Overall, its biocompatibility, biodegradability, toxicity, and other unique characteristics such as film efficiency, absorption, and antimicrobial activity make it a highly promising alternative to traditional packaging [11]. Chitosan can be readily modified chemically and physically to produce immune activation owing to an abundance of active functional groups on its molecular chain. Additionally, chitosan can be used in the promotion of wound healing [12], and bears antimicrobial and antifungal properties [13]. The flexibility and adaptability of chitosan provide a unique opportunity for the development of new antibacterial therapies and prevention of infectious diseases. For example, the adhesiveness of chitosan can be used for noninvasive mucosal vaccine vectors [14]. When chitosan is combined with wound dressings such as hydrogels, it can offer antimicrobial effects while promoting wound healing [15,16,17].

Natural gums have demonstrated the ability to inhibit a broad range of substances at low cost and low concentrations. A number of studies have been conducted that elaborate on the remarkable inhibition efficiency shown by natural gums in preventing corrosion of metal surfaces in aggressive environments, as previously mentioned in the background of the study. The long complicated polymeric structure of natural gums, which has various conjugately held pi electrons, as well as the large abundance of polysaccharides, oligosaccharides, and glycoproteins, which bear many electronegative functional groups, are responsible for the impressive inhibitory activity on metal surfaces (OH, COOH, NH_2_, etc.). The fruits, branches, and trunk of *Prunus dulcis* trees all produce a profuse gum termed almond gum. Arabinose (46.83%), galactose (35.49%), and uronic acid (5.97%) are the three main polysaccharides that comprise the majority of almond gum’s 92.36% constitution. There is only a small amount of protein (2.45%) [18]. The almond tree can produce almond gum, a natural adhesive with few jelly-like properties which acts as a cooler, while also providing a great texture and flavor to beverages. 

Almond gum is a water-soluble polysaccharide and its biocompatible characteristics are a result of its favorable hydrophilic and hydrogen bond characteristics. It has a number of advantageous applications, including food reinforcing, preservative, emulsifier, stabilizer, and is a source of dietary fiber. Moreover, it could be used as a component to increase a material’s physical and structural characteristics, such as hydration, oil-holding capacity, viscosity, texture, sensory properties, and shelf life [19]. Several types of edible material produced from almond gum, or mixtures of these bioplastics have recently been produced as a result of earlier studies. The lack of toxicity and excellent stiffness characteristics make it ideal for application in the industries of medicine, cosmetics, and food packaging [20]. In addition, almond gum is found to exhibit surface, interfacial and emulsification activities [21]. Although chitosan and other polymers have been utilized in multiple studies for food packaging applications, these two combinations are not described in any of them.

In recent years, the advancement of chitosan/almond gum composite’s industrial purpose has rapidly grown. It was determined that chitosan is a combination of all three, nontoxic, biodegradable, and has antimicrobial properties. It has many uses, mainly in the agricultural, food, and pharmaceutical industries. In this study, the fabrication and characteristics of CSA composites prepared using a solution casting are discussed. These properties are morphological, mechanical, barrier, thermal, and antimicrobial in nature. The aim is to investigate how loading almond gum affects the dissimilar physiochemical characterization of chitosan. The fabricated biocomposites demonstrate enhanced thermal, mechanical, and antimicrobial activities. Thus, it represents an ideal material for active food packaging applications.

## 2. Results and Discussion

### 2.1. FTIR Analysis

**Figure 1:**
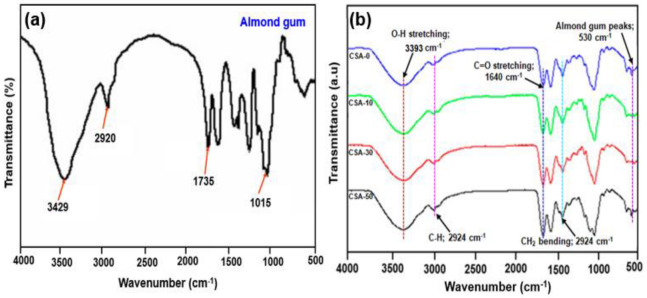
FTIR spectra of (**a**) almond gum; (**b**) chitosan, CSA-10.0, CSA-30.0, and CSA-50.0 composites.

The almond gum was subjected to FTIR analysis in order to determine it by seeking for functional groups in it (Figure 1a). The strong peak seen at 3429 cm^−1^ is a characteristic peak of O-H stretching vibrations. The asymmetric -CH_2_- functional groups correspond to the peak at 2920 cm^−1^. The distinctive peak at 1735 cm^−1^ is attributable to the carbonyl group’s stretching vibrations. The peak observed at 1015 cm^−1^ is because of bending of arabinosyl chains. These functional groups’ existence indicates the almond gum includes the prior stated compounds [22,23]. The FTIR spectra reveal interactions in the composites between carbonyl group (-C=O) of chitosan and the C-H stretching modes of almond gum. The peaks at 3393 and 2924 cm^−1^ in Figure 1b indicates the chitosan O-H stretching and C-H vibration, respectively. In accordance with the C-C stretching vibrations and C-O stretching modes, the peak at 1553 and 1415 cm^−1^ is placed. Stretching from C=O reaches 1640 cm^−1^. The 1430 cm^−1^ peak represent CH_2_ bending vibrations. In Figure 1b, the peaks at 530 cm^−1^ indicates the presence of almond gum stretching mode and all other peaks are due to the presence of soluble chitosan. The spectra of the chitosan/almond gum composites clearly show characteristic peaks corresponding to individual groups of chitosan. That is, the FTIR spectra of the CS composites show no change or shift in the characteristic peaks of pure chitosan with the addition of almond gum.

### 2.2. SEM Analysis of Chitosan and Its Composites

**Figure 2:**
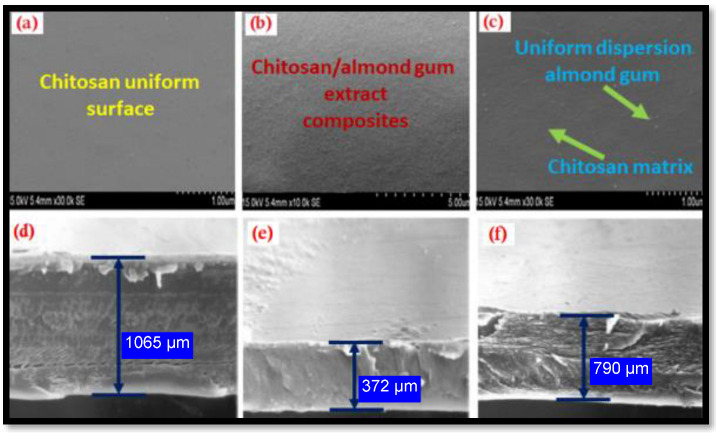
SEM micrographs of the (**a**) chitosan, (**b**,**c**) chitosan/almond gum extract composites; (**d**–**f**) the cross-section images of test samples. The chitosan and almond gum components are shown as green color arrows on the CSA composite film surface. The SEM cross-section images of the CSA films and chitosan film are represented by the blue-hued arrows.

The SEM micrographs and cross-section images of chitosan, almond gum and CSA composites are shown in Figure 2. The surface topology of CSA film compared to chitosan is observed and found that, these films observed that they do not show many changes in their microstructure which means their physical properties do not affect the concentration of almond gum on the film. Figure 2a shows that the microstructure of the neat CS film surface was smooth and uniform of CS. Meanwhile, the addition of 30 wt. % of almond gum to the CS film (Figure 2b) reduced the size of granules and improved the surface structure. As can be seen from Figure 2b, the film with 30 wt. % chitosan indicated uniform and smooth structure, and a few almond gum particles also appear on the film surface. However, when the amount of almond gum was increased to 50 wt. %, the almond gum was effusively blended with the CS. The surface structure from Figure 2c was smoother than Figure 2b, but more almond gum appeared on the surface of the film compared to that with 30 wt. % of almond gum. Since no shift is observed as a result of chitosan and almond gum interaction in composite materials, the existence of almond gum in the composite films displays outstanding reinforcing dispersion, without noticeable voids. This might be due to the even distribution of almond gum in the chitosan as well as good compatibility of the matrix.

### 2.3. TGA Analysis

**Figure 3:**
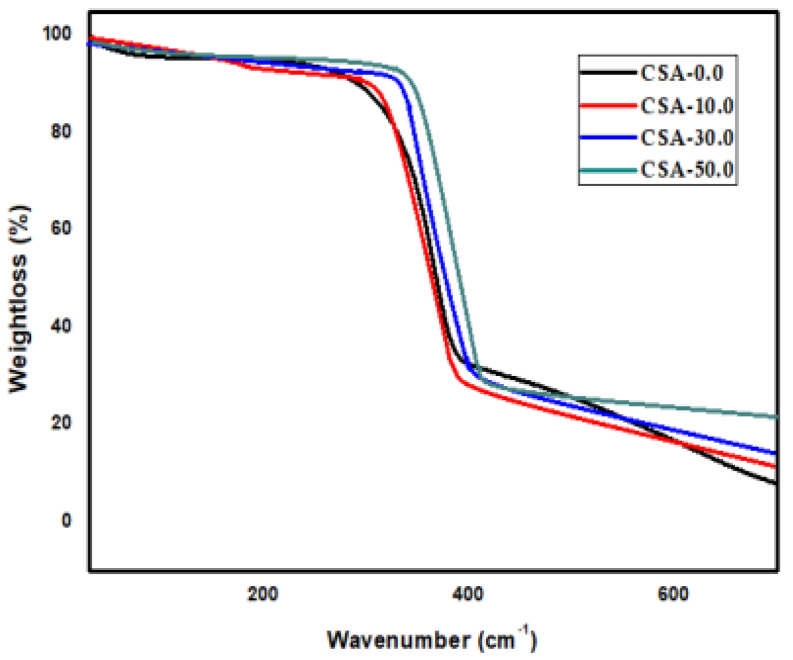
TGA curves of chitosan, and chitosan/almond gum composite samples.

The TGA curves of chitosan, chitosan/almond gum composites are shown in Figure 3. The weight loss at 50–160 °C is due to the moisture vaporization and the initial weight loss at 220–380 °C is due to the degradation of chitosan. Three weight losses were observed in the composites of chitosan and almond gum. The degradation of almond gum is the main reason of the 410 °C. The initial degradation of the CSA-10.0, CSA-30.0, and CSA-50.0 was further increased by the addition of 10.0, 30.0, and 50.0 wt. % of almond gum at 248, 295, and 380 °C, respectively. Chitosan/almond gum composite films were more stable thermally as pure chitosan, as shown by thermal stability changes in the film that were found as a result of the addition of almond gum in chitosan. After the incorporation of almond gums, the degradation curve shifted to higher temperatures; a higher almond gum loading led to a higher decomposition temperature. Additional residues were detected in the composites due to the good thermal stability of the almond gums.

### 2.4. Mechanical Test of Chitosan and Chitosan/Almond Gum Composites

**Figure 4:**
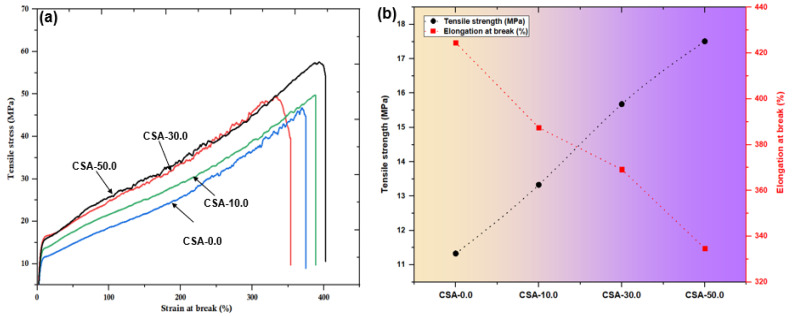
(**a**) Stress versus strain curves; (**b**) Tensile strength and elongation at break of CSA composites.

Figure 4a shows the stress vs. strain curves of chitosan and chitosan/almond gum composites. The effect of filler loading on the tensile strength, and elongation at break of almond gum-filled chitosan composites is presented in Table 1. The tensile strength of chitosan/almond gum film increased significantly from 15.56 to 22.83 MPa and so did the elongation at break, indicating a substantial drop from 424.5% to 334.7% with the addition of almond gum concentration from 10.0 wt. % to 50.0 wt. %. The significant increasement of 46.7% in tensile strength could be explained by the increase in chitosan as the almond gum concentration increased. When compared to the CS film, the chitosan/almond gum composite demonstrates improved tensile strength. This could be due to the interfacial bonding formed by the chitosan and almond gum. Figure 4b shows that the break of elongation for materials reinforced with chitosan and almond gum decreased from 424.5 to 334.7%. These results revealed that a 50.0 wt. % almond gum solution was too concentrated to produce a uniform and smooth film. The elongation at break reduced, which could be described by the concentration effect, in which the bonding between chitosan molecules increased as the almond gum concentration was increased to the maximum owing to the decomposition into the monomer units. The improvement in mechanical strength of chitosan/almond gum composites was probably due to the dispersion of almond gum in chitosan matrix. 

### 2.5. Film Thickness of Composite Film

**Figure 5:**
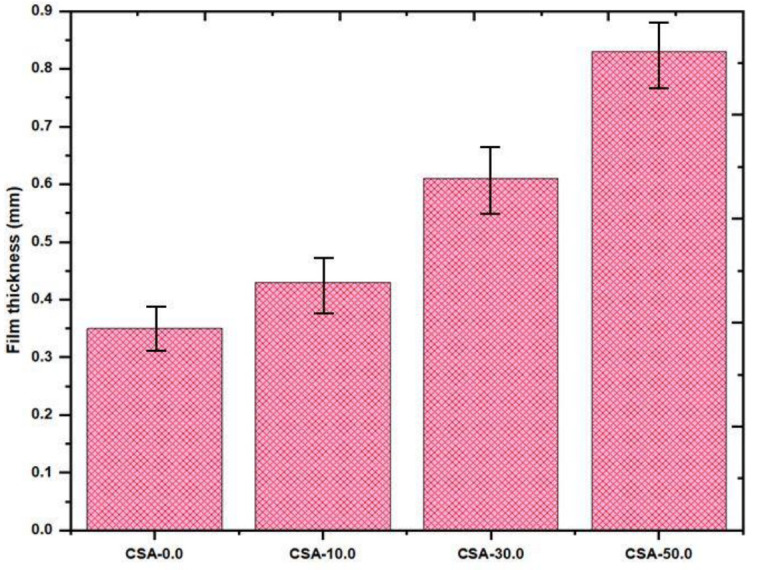
Film thickness versus film type. Error bars: ±2.05 SD.

Thickness of the different types of chitosan and its composite film values are shown in the Figure 5. Thickness of the films varied in the ranges between 0.35–0.83 mm. Pure chitosan film shows 23% of thickness while the almond gum reinforced chitosan/almond gum composites show 47% of thickness, this is due to the presence of almond gum which is producing a reaction in chitosan by departing the molecular chain and increasing the network, so this would probably lead to increase the thickness of the film. Standardization in thickness also plays an energetic role in film excellence. In addition, it might also influence the mechanical and barrier property.

### 2.6. Film Transparency

**Figure 6:**
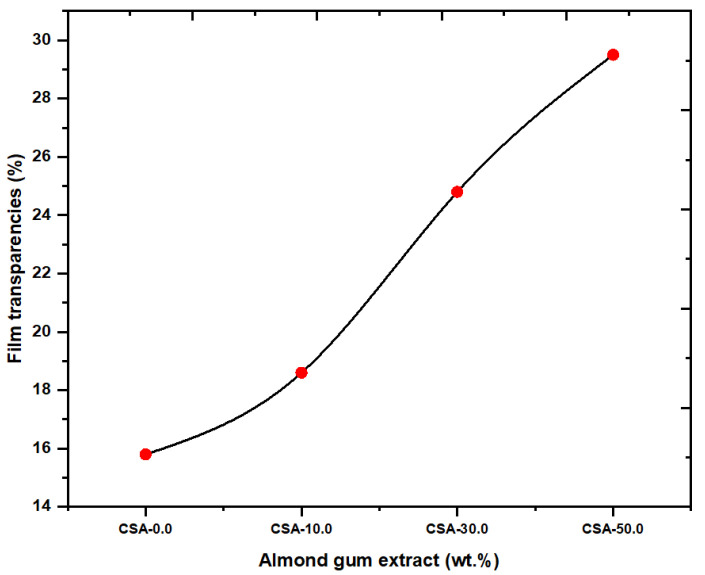
Film transparency versus film type.

The visual monitoring of the product’s excellence throughout preservation and aesthetic appeal to customers depends on the transparency of food packaging films. Figure 6 shows the transparency values of chitosan and chitosan/almond gum composites. The opacity value of chitosan is 15.8 A/mm. After the loading of 50.0 wt. % of almond gum, the opacity value increases to 29.5 A/mm. The maximum transparent of the four composites was CSA-50.0, which was also the opaquest. The aggregation of almond gum in the polymer affects the light scattering property and hence by adding the almond gum, the opacity level of the film was increased. Similar results in the transparency value for the chitosan composites were reported by Sanuja et al. [24]. Even if the values were decreased, the CSA film’s characteristic transparency suggests that adding almond gum could not have a significant effect on its appearance when used for packaging applications.

### 2.7. Swelling Property of Composites

**Figure 7:**
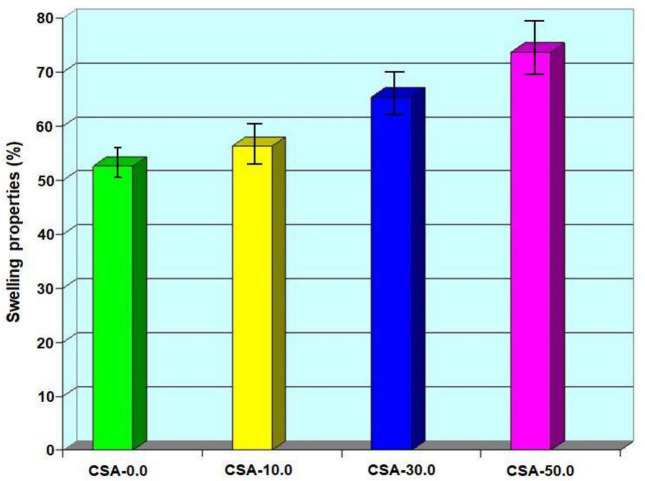
Swelling property versus film type. Error bars represent ± 5.01 standard errors.

The swelling property of many types of chitosan/almond gum composites are presented in Figure 7. The swelling property of the pure chitosan is determined initially, and then the swelling property of chitosan/almond gum composites is obtained and compared with the chitosan film. When the almond gum concentration is added to the chitosan film, the water absorbing tendency of the film decreases. It was observed that chitosan/almond gum composites show low swelling property (73.79%) when compared to chitosan film (52.67%). This is because the almond gum increases hydrophobicity of the film surface and reduces the water absorption of the chitosan matrix.

### 2.8. Water Solubility Test

**Figure 8:**
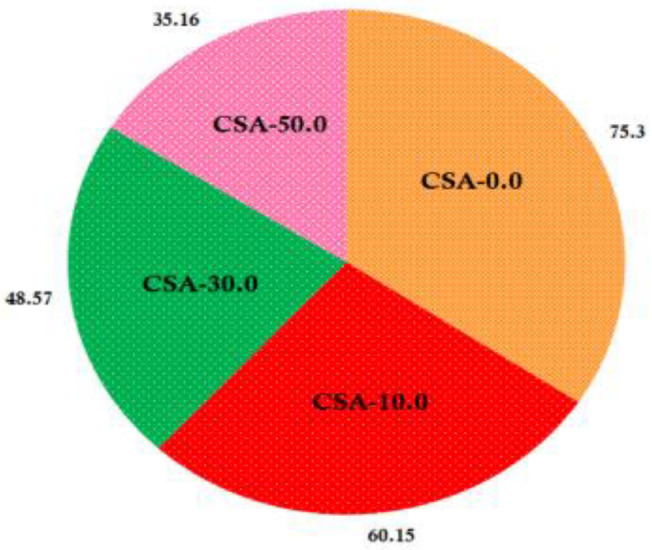
Water solubility versus film type.

Chitosan/almond gum composites solubility percentage in water is shown in Figure 8. This reveals the films solubility character toward the water in which the chitosan film shows higher (75.3%) affinity and better dissolves in water when compared to the chitosan/almond gum composites, since chitosan is hydrophilic in nature it absorbs water quickly and results in swelling. Incorporation of almond gum into the chitosan films shows considerably decreased solubility due to the presence of monosaccharides, (35.16%) in water this phenomenon is due to the effect of extreme hydrophobic nature of almond gum present in the surface of chitosan and thus the fabricated composite film shows low affinity toward water.

### 2.9. Barrier Property

The oxygen transmission rate is one of the most important properties of a packaging material. The OTR of chitosan/almond gum composites was determined, and the results are given in the Table 1. The value of OTR ranges from 90.6 to 32.9 cc/m^2^/24 h because almond gum provides both oligosaccharides and polysaccharides. The reinforcement of almond gum extract into the chitosan matrix improved the barrier properties of the composite films. The diffusion through the polymers happens due to the combination of two phenomena;

- Loading of filler decreases the area available for diffusion of gases.
- Increase in the distance that gaseous molecules must travel to cross the films.

It is believed that the lesser O_2_ permeability of composites containing (10.0, 30.0 and 50.0 wt. %) almond gum is attributed to well dispersion of the almond gum, which provides higher tortuosity of the diffusion path of oxygen molecules [25]. The measured WVTR values for the chitosan and its composites are listed in Table 2. The WVTR of the chitosan was significantly reduced by the incorporation of almond gum in the chitosan matrix. The WVTR values for chitosan, CSA-10, CSA-30 and CSA-50 were determined to be 22.1, 19.5, 15.9, and 11.6 g/m^2^/day, respectively. WVTR decreased with increase in concentration of almond gum in chitosan. The WVTR of chitosan/almond gum composites range from 22.1 to 11.6 g/m^2^/day, whereas the pure chitosan WVTR value is 22.1 g/m^2^/day. The decreases in permeability of composites are due to the presence of uniform dispersion of almond gum with increased percentages in the polymer matrix.

### 2.10. Antimicrobial Activity

**Figure 9:**
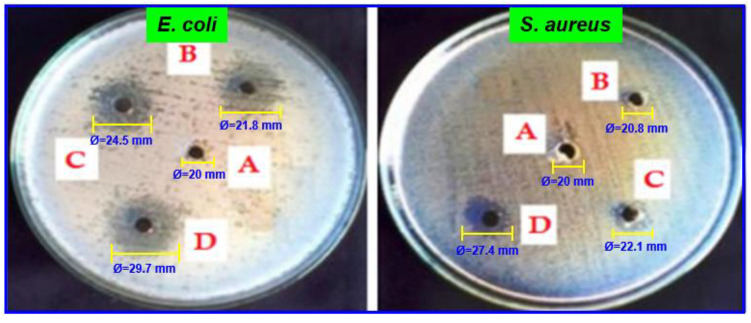
Antimicrobial activity: images depicting (**A**) chitosan film, CSA composites with (**B**) 10.0 (**C**) 30.0 and (**D**) 50.0 wt. % of almond gum.

Natural polymer-based composites have been studied recently as a polymeric matrix for incorporating antimicrobial agents [26]. The essential feature of food packaging materials is antimicrobial activity. *S. aureus* and *E. coli* were utilized as a test group for the antimicrobial activity of chitosan and chitosan/almond gum (CSA) composites using the zone of inhibition. The result values are listed in Table 3. There is no inhibitory zone against *S. aureus* and *E. coli* on the pure chitosan (CSA-0.0). Figure 9 exhibits the antimicrobial zones’ diameters in the CSA composites. The (CSA-50.0) composites showed strong antibacterial activity against *S. aureus* and *E. coli*, suggesting that almond gum could increase antimicrobial activity. The slightly reduced antimicrobial zone diameters for S. aureus comparison to those for *E. coli*. *S. aureus* give additional confirmation that the composites effects are real and significant against *S. aureus* bacteria. This was tested in order to evaluate that chitosan used to have a zone of inhibition activity in the CSA composite, and the results were in accordance with all earlier reports [27,28]. The zones of inhibition diameter of CSA composites are 20.0, 20.8, 22.1, and 27.4 mm against *S. aureus* and 20.0, 21.8, 24.5, and 29.7 mm against *E. coli* with loadings 0.0, 10.0, 3.0, and 50.0 wt. % almond gum, respectively. According to studies, the chitosan biodegradable polymer has strong antimicrobial activities against Gram-positive and Gram-negative microorganisms. Chitosan-based biodegradable plastics with some constituents have a maximal contribution and effective antimicrobial activity. So, this film can be used in food packaging applications to protect against the perishable products [29].

## 3. Materials and Methods

### 3.1. Chemicals and Materials

The crab shells were purchased from import and export companies (Chennai, India), and the almond gum was collected from Chennai, India. All the standards and chemicals used for the analysis were of analytical grade, whereas HPLC grade chemicals and standards were used in High-Performance Liquid Chromatography. Sodium hydroxide, hydrochloric acid, acetic acid (99%), and glycerol were purchased from Sigma Aldrich, India. All other chemicals and reagents obtained without further purification were used.

### 3.2. Collection and Purification of Almond Gum

As per Bhushette et al. [30], ethephon was packaged and injected in *Prunus dulcis* plants to lead to stress. Almond gum was purified to use the technique by Malsawmtluangi et al. [31] with the modification that ethanol instead of propanol was used for the precipitation. The non-soluble material was separated by centrifuging at 5000 rpm for 15 min after raw gum had been dissolved in water. After adding three volumes of ethanol to the precipitate, the precipitation was subjected to precipitated, centrifugation, and dried at 45 °C for 24 h to make dried gum. Following the addition of an enzyme, the gum’s color was observed to darken during the drying process. To resolve this issue, boiled ethanol was used on the raw gum prior purification in attempt to inactivate the enzyme. Before to use, purified almond gum was stored in an airtight container at a room temperature.

### 3.3. Synthesis of Chitosan Polymer

**Figure 10:**
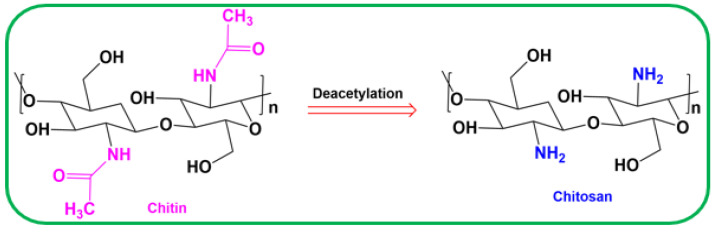
Deacetylation of chitin into chitosan and the associated chemical structures.

The chitosan was prepared by using method briefly mentioned in the literature [24]. In total, 250 g of crab shells were used, after being fully cleaned with water to eliminate all impurities, and then oven-dried at 110 °C. The shells were crushed to powder and been for 30 min at 60–70 °C with constant stirring while receiving an addition of 2% NaOH. Distilled water was used as a filter, and the process was repeated till the solution was colorless and clear. A 10 mL volume of HCl was added to the mixture as a confirmation. A total of 50 g of chitin was obtained after the product was dried at 60 °C overnight; this procedure is also known as demineralization. The repeat units of β-(1-4)-2-amino-2-deoxy-β-D-glucose are noticed in the acetamide group found in chitin and chitosan, a derivative of chitin (Figure 10). The functionality is commonly given by deacetylating chitin in basic media, which leads to the hydrolysis of the acetamide group to yield chitosan and acetic acid. The degree of deacetylation can be used as measurement unit for this mechanism. The extensive primary amine groups found in chitosan have been associated to all these properties.

### 3.4. Preparation of Chitosan/Almond Gum Extract Composites

The solution mixing and drop-casting method is used to produce the chitosan/almond gum composites [32,33]. The synthesized chitosan was pre-dried in oven at 60 °C for 4 h. The predicted amounts of almond gum (10.0, 30.0, and 50.0 wt. %) was then added to the 1% acetic acid solution in hot water, followed by magnetic stirring for 5 h at 50 °C. In order to fabricate chitosan/almond gum (CSA) composites, the stirring solution was cast onto a glass plate after 12 h followed by sonication for 30 min. After allowing the solvent evaporation and drying the composites for 48 h at 40 °C in an air oven, the chitosan/almond gum film were performed and the results. Table 4 indicates the composition percentages for the casting processes. The obtained samples were cut into 2.5 × 10.0 cm^−1^ pieces for tensile test.

### 3.5. Characterization

FTIR measurements were performed using a (Perkin Elmer spectrum RX1) instrument. The prepared chitosan, chitosan/almond gum extract composite films were subjected for scanning. Spectra were collected in the range of 4000–400 cm^−1^. Spectra were integrated by taking the area under the curve between the limits of the peaks of interest. SEM analysis was performed with a (JEOL, JSPM-100) electron microscope. Film samples were mounted on bronze stubs using a double-sided tape and then coated with a layer of gold (40–50 nm), allowing surface and cross-section visualization. All samples were examined using an accelerating voltage of 5 kV. Thermogravimetric analysis (TGA; TG-2009, Netzsch) was used to study thermal stability of chitosan, chitosan/almond gum extract composite films. The heating rate was 10 °C/min in N_2_ with the flow rate of 20 mL/min. The composite film samples were cut into strips that were 100 mm in length by 25 mm in width and fit to the tensile grips. The initial grip separation was set at 50 mm and the crosshead speed was set at 10 mm/min. The film specimens were mounted in the film extension grips of the testing machine and stretched at a rate of 50 mm/min until breaking. Tensile strength was expressed in MPa and was calculated by dividing the maximum load (N) by the initial cross-sectional area (m^2^) of the specimen. Thickness of the films was determined by the digital thickness measuring gauge to the nearest 0.001 mm. Presented values were taken for an average of at least five random locations of the films. The means were calculated and used in the determination of mechanical and physical properties of the films [30]. Transparencies of the composites were measured using UV spectrophotometer (Perkin Elmer, Lambda 35) by measuring the absorbance at particular wavelength of the different films. The films were cut into square pieces and inserted into the spectrophotometer test cell. An empty test cell was taken as the reference. The transparency of the films was calculated according to the formula.

Transparency = log (T wavelength range) X^−1^ (1)

where T wavelength range, is the transmittance at different wavelength of three many films and X is the thickness of the film (mm). According to this equation, when T has high values, it indicates lower transparency and higher degree of opacity [34,35]. Water content of the composite film was determined by measuring Weight gain of films, upon immersing the sample film in distilled water which is kept in Petri dish and allowed to remain there for 1 min then the wet weight is measured. The percent swelling ratio (%) of the CSA composites was calculated by the following equation [36].

Swelling (%) = (Wet weight − Dry weight)/Dry weight × 100(2)

The solubility of the CSA composite films (1 × 1 cm) was measured by immersing the pieces into 100 mL of distilled water and made to agitate in a mechanical shaker for 1 h at room temperature. Then the remaining samples were filtered and dried at 110 °C for few hours and it was weighed as final dry weight. The initial dry weight was measured before immersing it in water by drying at 110 °C [37]. Thus, the initial and final dry weight of the sample is obtained. By using the formulae, water solubility % is evaluated.

Solubility (%) = (Initial dry weight − Final Dry weight)/Initial dry weight(3)

The Oxygen transmission rate of the CS and its composites were measured by oxygen permeability tester (Noselabats, Italia) at 25 °C by using the standard of ASTM D-3985. The measurements were taken at three times at different places of the film and the average value was calculated. All specimens were conditioned at ambient conditions. A water vapor permeability analyzer (Mocon Permatran) was used to investigate the effect of composites on water vapor permeability of chitosan, and chitosan/almond gum extract composites. All the water vapor permeability tests were conducted at room temperature according to ASTM F-1249 standard methods. The test film was placed between two test cells. As the water vapor diffused through the test film, and it was carried by N_2_ to the detector in the upper chamber. N_2_ flow rate was set to 100 cm^3^ per minute. The data was recorded as water vapor transmission rate. The antimicrobial activities of the chitosan composites against *E. coli* and *S. aureus* were evaluated by using a zone of bacterial inhibition method (agar diffusion test) [38]. *E. coli* and *S. aureus* were activated by a constant temperature and humidity shaking incubator at 37 °C for 24 h. The cut circular samples (20 mm) were placed in a Petri dish and coated with 50 mL of the *E. coli* or *S. aureus* containing liquids. The plates were then incubated at 37 °C for 24 h, and the diameter of the inhibition zone was observed. 

### 3.6. Statistical Analysis

To determine the statistical significance of each result, ANOVA in SPSS 21 was used (IBM, New York, NY, USA). The data are given as the mean ± standard deviation. Statistical differences were analyzed using a one-way analysis of variance, and a value of *p* < 0.05 was determined to be statistically significant.

## 4. Conclusions

Chitosan and almond gum extract can be used to produce a composite film with desirable packaging characteristics. The CSA composite materials were successfully prepared by solution cast method. In chitosan, the concentration of almond gum extract varied from 10.0 to 50.0 wt. %. FTIR spectra demonstrated good interaction among the materials. Almond gum extract is dispersed uniformly in the chitosan matrix, according to the surface morphology owing to compatibility between the chitosan and almond gum extract. The addition of almond gum was found to increase the thermal stability of chitosan composites, which were directly influenced by almond gum dispersion and concentration. The tensile strength of the composite film was enhanced to 17.5 MPa by incorporating 50.0 wt.% almond gum into chitosan matrixes as compared to the tensile value of (11.3 MPa) pure chitosan. The most important packaging characteristic is opacity as it directly influences the product’s success regarding consumer appeal. It is a measure of the amount light prohibited from traveling through the packaging material, and high opacity represents a high level of optical characteristics [39,40]. The film solubility values showed that it has good solubility and low porosity. The swelling ratio of the films demonstrated its good water-uptake ability. Almond gum was discovered to improve tensile strength and film thickness when combined with chitosan, but had an opposite result on the film’s water solubility and barrier properties (OTR and WVTR). However, the antimicrobial activity associated with the addition of chitosan to the almond gum film shows excellent activity. These novel green composite films could find potential applications in transparent biodegradable food packaging.

## Figures and Tables

**Table 1:** Mechanical strength of chitosan and CSA composites.

Samples	Mechanical Strength	
	Tensile Strength (MPa)	Elongation at Break (%)
CSA-0.0	11.33 ± 1.06 ^b^	424.52 ± 1.85 ^a^
CSA-10.0	13.33 ± 2.08 ^a^	387.41 ± 2.22 ^c^
CSA-30.0	15.68 ± 1.87 ^c^	369.15 ± 2.90 ^c^
CSA-50.0	17.51 ± 0.75 ^b^	334.70 ± 0.60 ^b^

^a–c^: Different letters within the same column indicate significant differences among film samples (*p* < 0.05).

**Table 2:** OTR and WVTR properties of chitosan and chitosan/almond gum composites.

Samples	Barrier Properties	
	OTR (cc/m^2^/24 h)	WVTR (g/m^2^/day)
CSA-0.0	90.6 ± 2.05 ^b^	22.1 ± 0.71 ^a^
CSA-10.0	74.0 ± 0.98 ^a^	19.5 ± 1.22 ^c^
CSA-30.0	52.5 ± 0.61 ^c^	15.9 ± 0.99 ^c^
CSA-50.0	32.9 ± 1.95 ^b^	11.6 ± 1.62 ^b^

^a–c^: Different letters within the same column indicate significant differences among film samples (*p* < 0.05).

**Table 3:** Antimicrobial activity of chitosan and its composites against *S. aureus* and *E. coli*.

Samples	Antimicrobial Activity (Zone of Inhabitation in mm)	
	*S. aureus*	*E. coli*
CSA-0.0	20.0 ± 2.56 ^a^	20.0 ± 2.61 ^a^
CSA-10.0	20.8 ± 3.08 ^a^	21.8 ± 4.65 ^c^
CSA-30.0	22.1 ± 1.85 ^c^	24.5 ± 2.48 ^b^
CSA-50.0	27.4 ± 3.94 ^b^	29.7 ± 3.02 ^b^

Results are quoted as the mean ± standard deviation of three replicates. ^a–c^: Different letters within the same column indicate significant differences among film samples (*p* < 0.05).

**Table 4:** The formation ratio of chitosan and almond gum composites.

Chitosan/Almond Gum Composites (wt. %)	Samples Name
100.0–0.0	CSA-0.0
90.0–10.0	CSA-10.0
70.0–30.0	CSA-30.0
50.0–50.0	CSA-50.0

## Data Availability

Not applicable.
